# Nurses’ knowledge of and attitude toward postoperative patient-controlled analgesia (PCA) and the associated factors

**DOI:** 10.1186/s12912-024-01702-9

**Published:** 2024-01-05

**Authors:** Ying-Ru Chen, Cheryl Chia-Hui Chen, Wei-Wen Wu, Fu-Ing Tang, Ling-Chun Lu

**Affiliations:** 1 School of Nursing, College of Medicine, National Taiwan University, Taipei, Taiwan (ROC); 2 Department of Nursing, National Taiwan University Hospital, Taipei, Taiwan (ROC); 3 School of Nursing, National Yang-Ming Chiao Tung University, Taipei, Taiwan (ROC); 4 Second Degree of Science in Nursing, College of Medicine, National Taiwan Univeristy, 10051, No 1, Jen-Ai Road Sec. 1, Taipei, Taiwan (ROC); 5 Department of Nursing, National Taiwan University Hospital Yu-Lin Branch, Yu-Lin county, Taiwan (ROC)

**Keywords:** Attitude, Knowledge, Nurse, Pain, Patient-controlled analgesia

## Abstract

**Background:**

Postoperative pain control is pivotal for surgical care; it facilitates patient recovery. Although patient-controlled analgesia (PCA) has been available for decades, inadequate pain control remains. Nurses’ knowledge of and attitude toward PCA may influence the efficacy on clinic application.

**Purpose:**

The purpose of this study is to evaluate nurses’ knowledge of and attitude toward postoperative PCA and investigate the associated factors.

**Methods:**

This is a cross-sectional study. We enrolled registered nurses from a 2200-bed medical center in northern Taiwan within one year. The participants completed an anonymous self-reported PCA knowledge inventory and PCA attitude inventory. Data were analyzed descriptively and associated were tested using logistic regression.

**Results:**

With 303 participants enrolled, we discovered that nurses had limited knowledge of and a negative attitude toward PCA. Under half of the participants know how to set up a bolus dose and lockout intervals. The majority held misconceptions regarding side effect management for opioids. The minority agree to increase the dose when a patient experienced persistent pain or suggested the use of PCA. Surprisingly, participants with a bachelor’s or master’s degree had lower knowledge scores than those with a junior college degree. Those with 6–10 years of work experience also are lower than those with under 5 years of experience. However, the participants with experience of using PCA for patient care had higher knowledge scores and a more positive attitude.

**Conclusions:**

Although postoperative PCA has been available for decades and education programs are routinely provided, nurses had limited knowledge of and a negative attitude toward PCA. A higher education level and longer work experience were not associated with more knowledge. The current education programs on PCA should be revised to enhance their efficacy in delivering up-to-date knowledge and situation training which may convey supportive attitude toward clinical application of PCA.

## Background

With the continued increase in surgical volume and greater concerns regarding surgical quality, surgical care is now more critical than ever [1]. Postoperative pain control facilitates patient recovery and reduces postoperative functional impairment, which is crucial to adequate surgical care [2]. Patient-controlled analgesia (PCA) can result in higher patient satisfaction, stronger positive perceptions of situational control, lower preoperative anxiety, lower postoperative depression, and stronger pain relief; thus, it has been widely used for postoperative pain control since the 1970s [3, 4].

In PCA, a sophisticated microprocessor-controlled infusion pump is controlled by a patient-demand button and delivers a preprogrammed dose of opioids; the rationale for its efficacy is that a minimum effective analgesic concentration can dramatically decrease pain severity; the analgesic concentrations are varied and discrete, depending on the patients [5]. Although PCA has been in use for decades, the persistence of inadequate pain control requires expert attention [3, 4]. Despite the use of PCA, approximately 41% of patients experienced moderate to severe postoperative pain, and 80% of patients have experienced inadequate pain relief [2, 4]; the causes of these problems should be determined and resolved.

Pain has been defined by the International Association for the Study of Pain as an unpleasant sensory and emotional experience associated with, or resembling that associated with, actual or potential tissue damage [6]. Well pain control dependents on healthcare professionals understand the invisible pain reported by patients [7]. There are many barriers influence the outcome of patients’ pain management, including knowledge and belief of and attitude toward using analgesics whether healthcare profession or patients; communication between both of them; strict regulation of opioids and costs of interventions, etc. [7–9]. Nurses’ practice on pain management is closely related to these barriers, especially their knowledge and attitude [10, 11].

The knowledge and attitude toward pain management are also influenced by associated factors, such as age, gender, education level, and clinical care experience, but the influence remains controversy under different situations of pain management [10–13]. On the factor of clinical care experience, it often focused on working years but seldom mentioned about experience of using PCA for patient care [10, 12, 14]. Pervious study usually explored nurses’ knowledge of and attitude toward usage of single analgesia, such as non-steroid anti-inflammatory agents or opioids alone, but seldom mention about PCA [9, 10, 14]. There is a gap in the literature regarding nurses’ knowledge of and attitudes toward postoperative PCA and the associated factors, the investigation of these topics may enhance the work of nurses [10, 12, 15, 16].

### Purpose

The purpose of this study was to evaluate the levels of nurses’ knowledge of and their attitude toward postoperative PCA as well as to explore the factors associated with nurses’ knowledge and attitude.

## Methods

### Design and data collection

This study had a cross-sectional design and used convenience sampling. It was conducted in a 2200-bed medical center in northern Taiwan for one year. Participants with intent to join study were introduced by head nurse from five surgical intensive care units (SICUs) and nine surgical wards, including a neurological SICU, a cardiovascular SICU, a thoracic SICU, a general SICU, a traumatic SICU, three orthopedic surgery wards, two neurosurgery wards, a cosmetic surgery ward, an upper-gastrointestinal surgery ward, a hepato-cholecystic surgery ward, and a colorectal surgical ward. Before data collection, all participants provided written informed consent and understood the objectives, procedures, expected time commitment, and participants’ rights. Nobodies rejected the invitation. Thereafter, an anonymous self-report questionnaire was used to collect data. And one of each group of participants help to return the questionnaires and made sure completing all answers.

### Participants

Inclusion criteria of participants were register nurses servicing in surgical intensive care units or surgical wards with more than 3 months of work experience. Exclusion criteria was unable to complete the questionnaire.

### Instruments

The questionnaire consisted of a cover letter, sociodemographic characteristics sheet, PCA knowledge inventory, and PCA attitude inventory. The cover letter explained the research’s purpose and participants’ rights. The sociodemographic data sheet contained items pertaining to age, gender, education, duration of work experience, and experience of using PCA for patient care.

The PCA knowledge inventory was developed by Chu [17]. The inventory contains 20 items related to PCA knowledge, including the design principles of PCA, PCA setup, PCA side-effect management, and PCA administration. For each item, the possible responses were *true*, *false*, or *do not know*. Five points were allocated for correct answers. No points were allocated for answers that were incorrect or marked as unknown. Total scores ranged from 0 to 100 points. Participants who attained 60 to 100 points were deemed to have a high level of knowledge, whereas those who attained 59 points or fewer were deemed to possess limited knowledge. Content validity, face validity and construct validity were conducted in the original research [17]. Five experts were invited to establish the content validity, including four physicians and a pharmacologist. All of them had experience with research and education on pain management. They rated the inventory using a Likert scale (1–4, rating from “not relevant” to “high relevant”). To calculate the 4 points items, the research instrument’s mean scale content validity index was 0.82. Five nurses were invited to establish the face validity using a dichotomous scale (“clear or unclear”) and were asked to revise and provide clear statement when they rated the items as unclear. All of the terms in items were thought readable and clarify. On construct validity, two contrast group tests were conducted, including one group with nurses vs. anesthesia nurses and another group with anesthesia nurses vs. anesthesia physician. The discrimination in the groups were significant (*p <.001*). The reliability coefficient (Cronbach’s α) was 0.86 in this study, which indicates sufficient reliability.

The PCA attitude inventory was used to evaluate nurses’ attitudes toward pain assessment, management strategies for PCA, and administration of PCA [18]. The PCA attitude inventory is a 15-item scale that is scored using a 5-point Likert scale ranging from 0 (*never*) to 4 (*always*). A higher score indicated a more positive attitude toward PCA. The cutoff for a negative versus positive attitude for each item was 3 points. Total scores ranged from 0 to 60 points. Participants who scored 45 to 60 points were deemed to have a positive attitude toward PCA. Conversely, participants who scored 44 points or fewer were deemed to have a negative attitude toward PCA. Five experts were invited to establish the scale’s content validity. The same approaches of content validity, face validity and construct validity were conducted as the PCA knowledge inventory [18]. Eventually, research instrument’s mean scale content validity index was 0.97, all terms in items were readable and clarify, the construct validity was significant (p <.001). The reliability coefficient (Cronbach’s α) was 0.87 in this study, indicating sufficient reliability. The content validity index score was 0.97 and the reliability coefficient (Cronbach’s α) was 0.87, indicating sufficient reliability.

### Data analysis

The sample size required for logistic regression was estimated using G*Power software version 3.1 (odds ratio, 2; power, 0.95) [19]. It was established using z test, logistic regression, two tails, odds ratio 2.2, hypothesis of good knowledge with no experience of using PCA for patient care 0.1, power 0.8, ratio of experience and no experience of using PCA for patient care 0.5. The odds ratio was set on 2.2 according to the significant findings of predictor factors in the past study, which were around 1.7 and 2.8, and the mean was approximately 2.2 [14]. The number of participants roughly estimated 300 at least when churn rate 10% was considered.

The data were analyzed using SPSS 21.0 (IBM, Armonk, NY, USA). Descriptive statistics [percentage distributions, means, and standard deviations (SD)] were used to summarize the sociodemographic characteristics and inventory scores. On the basis of their scores for knowledge of PCA and attitude toward PCA, the participants were divided into two subgroups. Binary logistic regression analysis was used to investigate the influence of variables on nurses’ knowledge of and attitude toward PCA.

### Ethical considerations

The researchers fully complied with all the applicable regulations to protect the privacy of the participants, such as the Declaration of Helsinki and Computer-Processed Personal Data Protection Act, etc. This study was approved by the Research Ethics Committee of National Taiwan University Hospital (Approval No. 201603008RINA). All of written inform consents were obtained before data collection.

## Results

### Sociodemographic characteristics of participants

The sample consisted of 303 female nurses. Their sociodemographic characteristics are presented in Table 1. Majority of the participants were younger than 30 years old, held a bachelor’s degree, had work experience of less than 5 years, and had experience of PCA in patient care.

**Table 1:** Sociodemographic characteristics of participants (N = 303)

Variable	n (%)
Age (mean, SD: 31.40, 7.32)	
21–30 years old	175 (57.8)
31–40 years old	93 (30.7)
41–50 years old	29 (9.6)
≧ 51 years old	6 (2.0)
Education	
Junior college	17 (5.6)
Bachelor	272 (89.8)
Master and over	14 (4.6)
Duration of work experience (mean, SD: 7.62, 6.49)	
0–5 years	151 (49.5)
6–10	86 (28.4)
11–15 years	28 (9.2)
≧ 16 years	38 (12.5)
Experience of using PCA for patient care	
None	99 (32.7)
Yes	204 (67.3)

Abbreviations: SD: standard deviation; PCA: patient-controlled analgesia

### Nurses’ knowledge of PCA

The nurses had a limited level of knowledge of PCA. The mean PCA knowledge inventory score was 41.90 (SD, 15.71), and the scores ranged from 15 to 95. The correct response rates for the PCA knowledge inventory are presented in Table 2. Majority of the participants possessed a high level of knowledge on the design principles of PCA but limited knowledge on PCA administration. The participants had moderate degrees of knowledge regarding the PCA setup and PCA side-effect management.

**Table 2:** Nurses’ knowledge of PCA (N = 303)

Domains	No	Items	Correct response rates n (%)
Design principles of PCA	1	The design of PCA is informed by the concept of patient-demand analgesic administration. (O)	291 (96.0)
	2	The mechanism of PCA is the provision of a new analgesic to achieve a satisfactory analgesic effect and to maintain peak serum concentration. (X)	169 (55.8)
	3	The definition of bolus dose for PCA is the maximum dose reaching a moderate analgesic effect with the probability of moderate side effects. (X)	201 (66.3)
PCA setup	4	The common routes of administration for PCA include intravenous, epidural, and continuous subcutaneous injections (for terminal cancer patients and others). (O)	275 (90.8)
	5	The setup of the PCA pump includes the loading dose, bolus dose, continuous dose, lockout interval, and 4 h-limit dose, among other components. (O)	294 (97.0)
	6	The bolus dose of PCA should generally be set to half of the dose of intramuscular injection. (X)	118 (38.9)
	7	The suggested lockout interval of intravenous PCA is 30 to 60 min. (X)	133 (43.9)
PCA side effects management	8	The medication used in IV PCA should be nonopioid analgesics because the side effects of these medications are limited. (X)	69 (22.8)
	9	When a PCA overdose is suspected, the use of flumazenil as an antagonist should be considered to confirm the diagnosis. (X)	157 (51.8)
	10	Because of the opioid analgesics used in PCA, the most common side effect is addiction. (X)	55 (18.2)
	11	A respiration rate of less than five breaths per minute may be due to an overdose of PCA analgesics. (X)	238 (78.5)
	12	To prevent addiction to IV PCA, the best choice of PCA analgesics is NSAIDs. (X)	102 (33.7)
PCA administration	13	The goal of pain relief is achieved when the caregiver uses the PCA equipment directly. (X)	71 (23.7)
	14	The best advantage of PCA is that uncooperative patients can be prioritized for PCA administration. (X)	25 (8.3)
	15	Although patients may use PCA by themselves, the nurse should educate the patient as much possible to reduce the use of PCA to prevent overdose and side effects. (X)	133 (43.9)
	16	The intended analgesic effect of PCA is that patients do not feel pain at all and their pain score is 0 out of 10 on the visual analogue scale; otherwise, it is necessary to increase the dose. (X)	25 (8.3)
	17	It is acceptable to extend the use of PCA for 14 days of a patient requests it when the analgesic effect of PCA is satisfactory. (X)	63 (20.8)
	18	The same class of IV PCA analgesics should be administered to achieve an analgesic effect when the effect of IV PCA is unsatisfactory. (X)	54 (17.8)
	19	The analgesic used in IV PCA and epidural PCA is the same; therefore, it is possible to administer the epidural PCA analgesic directly via the intravenous route. (X)	80 (26.4)
	20	Pain is a subjective feeling; thus, nurses’ knowledge of pain and their options for treatment cannot influence the time and dosage of analgesic administration and the outcomes of postoperative pain control. (X)	104 (34.3)

Abbreviations: PCA: patient-controlled analgesia; IV: intravenous; NSAIDs: nonsteroidal anti-inflammatory drugs; SD: standard deviation

Over half of the participants understood the design principles of PCA, particularly those pertaining to patient demand (correct response rate, 96.0%). Regarding PCA setup, under half of participants were familiar with how to exchange a bolus dose from an intramuscular to an intravenous route (correct response rate, 38.9%) and the suggested lockout interval of intravenous PCA (correct response rate, 43.9%). Regarding side-effect-management, most of the participants tended to avoid the use of opioids because of mistaken beliefs regarding its side effects, such as miscellaneous side effects (correct response rate, 22.8%), the high potential for common side effects (correct response rate, 18.2%), and the prevention of addiction (correct response rate, 33.7%). On the administration of PCA, most participants resisted the idea of on-demand patient usage (8.3–23.7%); had misconceptions relating to the goal of pain control (correct response rate, 8.3–43.9%), duration of usage (correct response rate, 20.8%), choice of effective analgesics (correct response rate, 17.8%), and advisable routes (correct response rate, 26.4%); and lacked confidence in their ability to influence patient usage of PCA (correct response rate, 34.3%). Only 8.3% of the participants agreed to increase the dosage when patients continued to experience pain.

### Nurses’ attitude toward PCA

The nurses held relatively negative attitudes toward PCA. The mean PCA attitude inventory score was 41.35 (SD, 6.07), and range of scores was 26 to 59. The positive responses for PCA attitude inventory scores are presented in Table 3. Most of the participants had a positive attitude toward pain assessment and PCA management strategies but an ambivalent attitude toward the administration of PCA.

**Table 3:** Nurses’ attitude toward PCA (N = 303)

Domains	No	Items	Positive respondents n (%)
Pain assessment	1	You can conduct a pain assessment when you care for patients experiencing pain.	297 (92.1)
	2	You can accept patients’ subjective judgements when they complain of pain.	267 (88.1)
	3	You can evaluate the severity of pain, vital signs, consciousness level, mental status, and history of allergy to opioids before giving patients use of PCA.	241 (79.5)
Management strategies for PCA	4	You would not ask patients to endure their pain because of concerns regarding side effects (i.e., overdose) when patients complain that opioid analgesics are ineffective.	91 (30.0)
	5	You re-evaluate patients’ pain and discuss adjustments of the type, route, and dosage of analgesics with the doctor when a patient complains that analgesics are ineffective.	268 (88.4)
	6	You evaluate patients continually and discuss the adjustment of analgesic doses with doctors in a timely manner for high-risk patients, such as individuals with poor renal function, obesity, or obstructive sleep apnea, and for those older than 70 years old, who are more likely to experience side effects when using opioid analgesics.	199 (65.7)
	7	You agree that analgesic-relevant training helps you understand how to use analgesics and evaluate the effects and adverse side effects of analgesics.	201 (66.3)
	8	You educate patients using PCA on the adverse side effects of opioids and how to use the PCA device.	244 (80.5)
Administration of PCA	9	You agree that PCA-knowledge-relevant training helps you understand how to educate patients to use PCA and address the problems resulting from PCA.	179 (59.1)
	10	When patients experience pain, you encourage them to push the button for PCA administration immediately and tell them that it will not result in an overdose.	234 (77.2)
	11	You agree that PCA is a safe mode of medication administration and that it can relieve patients’ pain effectively.	226 (74.6)
	12	You agree that PCA is easy to use and to troubleshoot.	146 (48.2)
	13	You agree that PCA can help to reduce the dose of analgesics required and the occurrence of side effects.	150 (49.5)
	14	You evaluate patients’ needs and ask doctors to recommend the use of PCA to patients.	131 (43.2)
	15	You believe that the various routes and medication types for administering PCA are a potential source of error for nurses during analgesia administration.	169 (55.8)

Abbreviations: SD: standard deviation; PCA: patient-controlled analgesia

Most of the participants had confidence in patients’ pain assessment when they cared for patients experiencing pain (positive respondents, 79.5–92.1%). Regarding PCA management strategies, 30% of the participants stated that they would not ask patients to tolerate pain to prevent opioid overdose. Regarding the administration of PCA, under half of the participants agreed that PCA is easy to administer and to troubleshoot (positive respondents, 48.2%), that PCA usage has benefits for pain control (positive respondents, 49.5%), or asked the doctor to recommend PCA usage in response to patients’ needs (positive respondents, 43.2%).

### Factors associated with nurses’ knowledge of and attitude toward PCA

The factors associated with nurses’ knowledge of and attitude toward PCA are presented in Table 4. Higher education level and 6 to10 years of work experience were negatively associated with knowledge. Experience of using PCA for patient care was a positive predictor of knowledge and attitude.

**Table 4:** Factors associated with nurses’ knowledge of and attitude toward PCA

Variables	PCA knowledge						PCA attitude					
	*β*	OR		95% CI			*β*	OR		95%CI		
Age (years old)												
21–30	-						-					
31–40	2.388	10.892		0.295	-	402.161	0.069	1.071		0.108	-	10.599
41–50	2.843	17.170		0.727	-	405.300	0.144	1.155		0.216	-	6.165
51 and more than	1.514	4.543		0.393	-	52.508	−0.175	0.839		0.302	-	2.332
Education												
Junior college	-						-					
Bachelor	−2.721	0.066	*	0.005	-	0.917	−1.446	0.236		0.041	-	1.341
Master	−3.000	0.050	**	0.011	-	0.220	−0.909	0.403		0.121	-	1.346
Duration of work experience (years)												
0–5	-						-					
6–10	−3.736	0.024	*	0.001	-	0.605	0.540	1.716		0.327	-	8.994
10–15	−2.232	0.107		0.007	-	1.725	0.509	1.664		0.436	-	6.352
16 and more than	−2.126	0.119		0.013	-	1.125	0.452	1.571		0.686	-	3.597
Experience of using PCA for patient care	1.061	2.889	*	1.120	-	7.453	1.340	3.821	***	1.907	-	7.657

Abbreviations: PCA: patient-controlled analgesia; 95% CI: 95% confidence interval

* *p* <.05; ** *p* <.01; *** *p* <.001

Nurses with a higher education level had more limited knowledge of PCA. Nurses with a bachelor’s degree scored 2.721 points lower than those with junior college [odds ratio, 0.066; 95% confidence interval (CI), 0.005–0.917], and nurses with a master’s degree scored 3 points lower than those with junior college (odds ratio, 0.05; 95% CI, 0.011–0.22). Nurses with 6 to10 years of work experience scored 3.736 points lower than those with work experience of less than 5 years (odds ratio, 0.024; 95% CI, 0.001–0.605). Nurses with experience of using PCA for patient care scored 1.061 points higher on PCA knowledge than those with no experience (odds ratio, 2.889; 95% CI, 1.120–7.453) and scored 1.34 points higher on PCA attitude than those with no experience (odds ratio, 3.821; 95% CI 1.907–7.657).

## Discussion

The objectives of this study were to evaluate nurses’ knowledge of and attitude toward postoperative PCA and to explore the factors associated with nurses’ knowledge and attitude. The findings of this study support the notion that nurses have limited knowledge of and a negative attitude toward PCA. Under half of the participants had knowledge of how to exchange a bolus dose from an intramuscular to an intravenous injection and the suggested lockout intervals. The majority held misconceptions regarding side-effect management for opioid analgesics. Notably, only 8.3% of participants would agree to increase the dose when a patient continued to experience pain. Over 90% of participants felt they could conduct pain assessments, but under half of the participants suggested the use of PCA. The predictors of nurses’ poor knowledge were found to be higher level of education and 6 to10 years of work experience. A predictor of favorable knowledge and attitude was experience of using PCA for patient care.

The characteristics of the participants in this study were typical of nurses in Taiwan. According to statistics from the Health Information Network of the Ministry of Health and Welfare in Taiwan, the vast majority of nurses are female (97.1%), the mean age of nurses is 30.84 years, and the average work experience of a nurse is 6 to 7 years [20]. In this study, all of the participants were female, their mean age was 31.4 (SD, 7.32) years, and their average work experience was approximately 7.62 (SD, 6.49) years.

Nurses’ knowledge of pain management has been addressed in the literature, but it remains considerably limited [10, 12, 21]. Similar findings have been reported on how nurses regard postoperative pain management and cancer pain management [10, 12, 22]. In this study, most nurses had limited knowledge of PCA and the correct response rate for the PCA knowledge inventory was approximately 41.9%. The correct response rate among nurses for knowledge of postoperative pain management was 45.7% in Jordan [10]. The correct response rate among nurses for knowledge of cancer pain management was approximately 51.5% in a study analyzing data from Canada, the United States, Italy, Japan, and Spain [12]. The correct response rates for nurses’ knowledge of cancer pain control were reported to be 50% in Ethiopia, 40% in Brazil, and 36.4% in Palestine [9, 14, 23]. The limited knowledge of nurses regarding management of various situation of pain management appears to be a common issue; this should be considered a weak point of global concern on nursing care.

It is crucial to investigate the precise nature of deficiencies in the knowledge of nurses. Such investigations can inform future training programs and the empowerment of nurses. Studies have investigated nurses’ limited knowledge of cancer pain control and focused on the use of opioids [13, 22]. Scholars explored the precise nature of limited knowledge of postoperative pain control, and they focused on pain assessment and route, frequency, and the time and side effects of administrating opioids [10]. In the present study, most of the nurses understood the design principles of PCA, especially those relating to patient demand. However, the majority of them avoided the use of opioids; resisted patient demands; had misconceptions regarding the goals of pain control, duration of usage, choice of effective analgesics, and advisable routes; and lacked confidence to influence patients’ usage of PCA. In the other word, nurses may understand the design principles of PCA, which are based on patient demand, but they do not apply these principles in clinical practice, which may be misconceptions regarding using PCA to care patients.

Regarding PCA setup, under half of the participants were familiar with the exchange of bolus dose between intramuscular and intravenous routes and the suggested setup of lockout intervals for intravenous PCA. Regarding PCA side-effect management, most participants tended to avoid the use of opioids because of mistaken beliefs regarding their side effects, such as high potential for side effects and the prevention of addiction. To comparing the findings with previous studies, all of them indicated opioids and side-effect management were equally confusing to nurses whether the PCA was for postoperative pain control or cancer pain control [9, 15]. A cause of the conflict may associate with strict usage of opioids, which is the consequence of overestimating side effects of opioids [9, 15]. According to a statistic result from database of Centers for Disease Control and Prevention in Unite Stats between 2006 and 2016, the ratios of opioid use disorder or overdose were 0.07–0.19% and prescribed medicine with less than 90 morphine equivalence doses daily in any months was safer than the non-prescribed [24]. Thus, an emphasis on patient demand, the prevalence of opioid use disorder or overdose, details regarding PCA setup, indications on the usage of PCA and clinical judgement for managing the side effects of PCA should be included in the program of nursing education. Training in such matters may increase nurses’ confidence in practice.

Nurses’ negative attitude toward pain management has been reported elsewhere [15, 22]. One study explored oncological nurses’ attitude; the nurses agreed that patients were the most reliable source of information regarding their own pain and that patients can be offered relief from severe pain, but they disagreed on the usage of placebo [22]. In this study, most nurses also held a negative attitude. They trusted the pain assessments of the patients themselves. Usage of a placebo for pain control is no longer recommended practice [6]. Regarding the administration of PCA, some negative attitudes are a cause for concern, for example, the view that PCA is troublesome and unbeneficial. Such beliefs may lead to nursing practice errors and hesitation to recommend the usage of PCA. The findings may reflect the need for PCA usage training programs for nurses who do not favor the use of PCA and consider it to have limited benefits. The definition of standard protocols for PCA administration should be an integral component of future training programs.

An interprofessional consensus regarding core competencies for pain management should encompass the multidimensional nature of pain, pain assessment and measurement, management of pain, and the contexts of pain management [25]. The primary focus of this study was postoperative pain control, and therefore the multidimensional nature of pain was not explored. This study discovered that majority of the participants had a positive attitude toward pain assessment but also limited knowledge of and a negative attitude toward the strategies and contexts of pain management. A qualitative study suggested nursing administration of pain control should involve patients’ perceptions which reported patients’ pain relief was improved when nurses provided information individually, repeated information to allow time for patients to overcome resistance related to dysfunctional beliefs and fear, facilitated patient involvement in pain control strategies, and provided tailored person-centered education [26]. The design of training programs should include clinical judgement about when PCA usage is appropriate. Situation training may be able to input in the program of nursing education.

In the literature, factors associated with nurses’ knowledge of and attitude toward pain management include age, gender, education level, and training and care experience [10, 12, 15]. Impactions of the factors on nurses’ knowledge and attitude in different situation of pain management remain inconsistence. Some studies have reported that age, education, and duration of work experience did not significantly influence nurses’ knowledge and attitude [9, 14, 22]. In some studies, oncological nurses with a master’s degree and longer work experience had better knowledge compared with those with a bachelor’s degree and less work experience [9, 23]. Another study reported that medical–surgical nurses with a postgraduate level of education and work experience of 5 to 10 years had greater knowledge and a more positive attitude toward pain management [15]. The present study discovered that a higher education level and work experience of approximately 6 to 10 years were predictors of nurses’ poor knowledge, but experience of PCA usage for patient care was a predictor of nurses’ greater knowledge and positive attitude. These associated factors exert different influences on various types of pain management. Notably, some nurses were found to have limited knowledge and a negative attitude despite having received training on PCA or longer work experience. There is a bold assumption, the experience of using PCA for patient care may the more important associated factor than the high-level education or longer work experience on nurses’ knowledge of and attitude toward postoperative PCA. According to studies on adult learning, motivation is crucial for promoting adult learning and ensuring positive outcomes of learning; trail-on-error also is a very important process to learn, which may provide a concrete experience from knowing to how to do [27, 28]. The demands of prepared knowledge and skills of daily practice should be the motivation of nurses’ learning, which also supported the gap of nurse education by a meta-analysis study [21].

The limitation of this study is categories of impacted factors on patients’ pain control outcome only focused on nurses’ knowledge and attitude because interest and motivation of research was focus on nurses’ reflection. The generalizability of findings in this study restricted on design of a cross-sectional study and data collection from a single huge medical center. Further randomized clinic trail to validate effectiveness of education program on post-operative pain management with up-to-date knowledge and situation training will be worth to comply. The strengths of this study are to expose the education components of nurses’ knowledge, which illustrate in implications for nursing practice.

### Implications for nursing practice

Current PCA training programs and college curriculums must be revised to enhance their efficacy in delivering up-to-date knowledge and conveying a supportive attitude toward clinical application of PCA. Regarding the clinical relevance of these findings, this study supports future training programs for PCA that emphasize patient demand, details on PCA setup, the standard protocol for operating PCA pumps, indications on PCA usage, and clinical judgement for managing the side effects of PCA. Situation training may be one of the considered methods on the program.

## Conclusion

The contributions of this study to nursing were to support nurses had limited knowledge of and a negative attitude toward postoperative PCA despite to nurses had higher level of education, thus current educational programs should be redesigned to address this issue; to explore longer working years on clinical experience did not guarantee adequate knowledge and a positive attitude toward postoperative PCA, but experience of using PCA for patient care on clinical experience may be a key factor motivating nurses to learn about PCA and building a concrete bridge between knowledge and clinical practice, thereby enhancing their knowledge and promoting a positive attitude. In the conclusion, the current education programs on PCA should be revised to enhance their efficacy in delivering up-to-date knowledge and situation training which may convey supportive attitude toward clinical application of PCA.

## Data Availability

Data of all collected participants are available to share after deidentification.

## Abbreviations

PCA: patient-controlled analgesia
